# Is breastfeeding protective against the development of asthma or wheezing in children? A systematic review and meta-analysis

**DOI:** 10.1186/1710-1492-7-S2-A11

**Published:** 2011-11-14

**Authors:** Elinor Simons, Sharon D Dell, Joseph Beyene, Teresa To, Prakesh S Shah

**Affiliations:** 1 Child Health Evaluative Sciences, Hospital for Sick Children, Toronto, ON, Canada, M5G 1X8; 2 Division of Respiratory Medicine, Hospital for Sick Children, Toronto, ON, Canada, M5G 1X8; 3 Population Health Sciences, Hospital for Sick Children, Toronto, ON, Canada, M5G 1X8; 4 Clinical Epidemiology and Biostatistics, McMaster University, Hamilton, ON, Canada, L8S 4K1; 5 Division of Neonatology, Mount Sinai Hospital, Toronto, ON, Canada, M5G 1X5

## Background

Although breastfeeding is strongly recommended for its many benefits, the association between breastfeeding and childhood asthma development remains controversial. Our objective was to systematically review and meta-analyze the association between physician-diagnosed asthma (PDA) or wheezing development and exclusive or any breastfeeding.

## Methods

Prospective cohort studies of preschool (4-6 years) and school-aged (7-9 years) children were identified from Medline (1948-June 2011) and Embase (1980-June 2011). Breastfeeding exposure for at least the first 3-4 months of life was defined as exclusive (breast milk as the only source of nutrition) or any (breast milk included in the diet). Outcomes were parent-reported PDA or wheezing. Risk of bias in included studies was assessed using the Newcastle-Ottawa scale. Data were analyzed using the Revman software package and adjusted odds ratios were meta-analyzed using random-effects models.

## Results

Ten studies enrolling 35,411 participants were included. Decreased odds of PDA or wheezing development at ages 7-9 years were identified for those who received exclusive breastfeeding [adjusted odds ratio (OR) 0.69, 95% confidence interval (CI): 0.58-0.83] and any breastfeeding (OR 0.53, 95% CI: 0.41-0.68) and at ages 4-6 years for those who received exclusive breastfeeding (OR 0.75, 95% CI: 0.61-0.93). Among the clinically-heterogeneous studies with outcome assessment at ages 4-6 years, any breastfeeding did not change the odds of PDA or wheezing (OR 1.08, 95% CI: 0.76-1.54).

## Conclusions

Exclusive or any breastfeeding for at least the first 3-4 months of life was associated with lower odds of PDA or wheezing in children, strengthening support for the current breastfeeding recommendations.

